# Progress towards a leprosy-free country: The experience of Oman

**DOI:** 10.1371/journal.pntd.0006028

**Published:** 2017-11-20

**Authors:** Salah T. Al Awaidy

**Affiliations:** Office of H.E. of Health Affairs, Ex EPI National Manager and Director of Communicable Diseases and Control, Ministry of Health, Muscat, Oman; London School of Hygiene and Tropical Medicine, UNITED KINGDOM

## Abstract

**Introduction:**

The World Health Organization (WHO) released the Global Leprosy Strategy 2016–2020 towards a leprosy-free world. The author described the progress made towards the elimination of leprosy and suggested recommendations for the acceleration towards a Leprosy-free country according to WHO laid out criterion.

**Methodology:**

Case record review of Leprosy patients managed between the years 1992 to 2015 were registered and analyzed. Data were collected from annual reports of the Ministry of Health including demographics, classification of leprosy new cases, relapse, childhood, grades of disability (GD) and multidrug therapy (MDT) completion rates.

**Results:**

Leprosy prevalence rate declined from 1.64 to 0.09 per 10,000 population during the period 1992 and 2015 (p<0.0001). Between 2005 and 2015, 77 patients were diagnosed with Leprosy as per definition and 75/77 (98%) had smear or biopsy positive. Of these, 53 (69%) cases were among foreign-born (non-national) (p<0.003) and 19 (25%) were among women. Most of the leprosy cases were notified in Muscat governorate 29 (38%) and among patients between 25–44 years of age 41 (53%), followed by ≥45 years 29 (38%) and 6 (8%) were children age ≤ 14 years. Multi-bacillary (MB) cases reported 60 versus 17 for Pauci-bacillary (PB) (p< 0.01), while MB was highest among both nationals (83%) and foreign-born (75%).

MDT completion rate was 100% and no relapse cases were notified among nationals. The rate of new patients diagnosed with leprosy related disability was 2.3 per million population, and grade 2 disability (G2D) rate among nationals was 0.9 per million population. No disability was recorded among women or children less than 14 years within the nationals group from 2013. Almost all the foreign-born patients didn’t complete their treatment in Oman as they left the country shortly after diagnosis of leprosy due to a very short term contract, discretionary employment practices by the employers and prefer to go home to complete their treatment.

**Conclusion:**

Oman has met the elimination goals and made great strides towards becoming a leprosy-free country. However, challenges such as improving surveillance system efficiency and sensitivity for detecting timely leprosy cases, as well as foreign-born workers are still a major concerns.

## Author summary

Leprosy is a chronic infectious disease caused by *Mycobacterium Leprae* that involves many body organs but mainly skin, peripheral nerves and mucous membranes and occasionally other organ systems that affects equally all races, ages and both sexes. While most individuals exposed to an infectious case of leprosy become infected, only less than 5 percent of those infected develop the disease and subsequent disability. In Oman by World Health Organization Standard’s, Leprosy has nearly been eliminated as a public health problem over the past two decades. We are however, striving to meet the new three pillars set by WHO to achieve a completely leprosy free country. The three main strategies employed include: strengthening government ownership, coordination and partnership to stop leprosy and its complications; and to stop discrimination and promote inclusion [1–3]. We hope that through fervent efforts aim towards Oman having zero leprosy cases by WHO standards in the very near futures.

## Introduction

In 1991, the World Health Assembly of the World Health Organization adopted a global resolution requesting all the member states for leprosy elimination as a public health problem by the year 2000. The World Health Organization (WHO) aimed for leprosy elimination to decrease the prevalence to <1 leprosy case per 10,000 population [1, 2].

Further, in April 2016, the Global Leprosy Strategy 2016–2020 released by WHO aimed at “Accelerating towards a leprosy-free world”. The strategy was built around three pillars, firstly strengthening government ownership, secondly coordination and partnership to stop leprosy and its complications; and lastly to stop discrimination and promote inclusion. In endorsing the global strategy, three key targets have been agreed upon by global national programs including (i) zero grade-2 disabilities (G2D) among children diagnosed with leprosy; (ii) the reduction of new leprosy cases with G2D to <1 case per million population; and (iii) zero countries with legislation allowing discrimination on the basis of leprosy [1, 3].

In 2016, world widely an estimated 174,608 leprosy cases were notifying receiving multidrug therapy (MDT), accounting to a prevalence rate of 0.29 per 10,000 population, a decrease from 0.32 per 10,000 in 2014 [3]. Globally, there has been an impressive progress towards achieving the targets of Global Leprosy Strategy, out of total member states, 39 member states registered zero children with G2D; 50 have a G2D rate among new cases of <1 per million population; and 67 have no legislation adopted yet that allows discrimination among patients [4].

WHO Eastern Mediterranean Region, reported an estimated 8,495 registered leprosy cases representing approximately 15% of the global leprosy burden however, some member states, leprosy is still considered a national burden. These include Egypt, Yemen, Iran and Qatar [5].

### Leprosy program in Oman

National leprosy program (NLP) in Oman was launched in 1981 along with the tuberculosis (TB) program in collaboration with central leprosy center unit at Al Nahdha Hospital (national tertiary care for the dermatology), aiming at reducing the morbidity and disability. Oman also has established an independent joint TB and leprosy technical expert committee to review the status of leprosy elimination, later the committee functions were integrated into the national communicable diseases committee, aiming on provides advice on corrective actions for achieving and maintaining successful leprosy-free country.

Furthermore, the program has been integrated into the Primary Health Care (PHC), secondary and tertiary care services provided by the Ministry of Health (MOH) and non-MOH institutions.

All leprosy-related activities, including surveillance and laboratory testing of suspected leprosy cases, are financially supported by the MOH and provided free of charge in government health institutions.

The program strategy is being implemented at several levels, the first at primary health care (PHC) level i.e. in dermatology clinics where cases are notified. Thereafter, the suspected case is referred to the nearest dermatology clinics for finalization and confirmation of diagnosis post confirmation follow-up is done and the assigned dermatologist usually administers MDT.

The national guidelines were developed in 1998, spelled out the policy and the package of care to be delivered through leprosy strategy. The strategy included case notification, pre-and post-test counseling, client flow, skin biopsy sample flow and roles and responsibilities of the health team at primary health care (PHC), dermatology clinics, follow-up and flow of patient at different care centers. Furthermore, contact tracing was also spelled out, has evolved and is widely implemented [6].

In this report, the author describes the progress made towards a Leprosy-free nation and the challenges ahead to achieve and maintain this target in Oman.

## Methods

**Fig 1:**
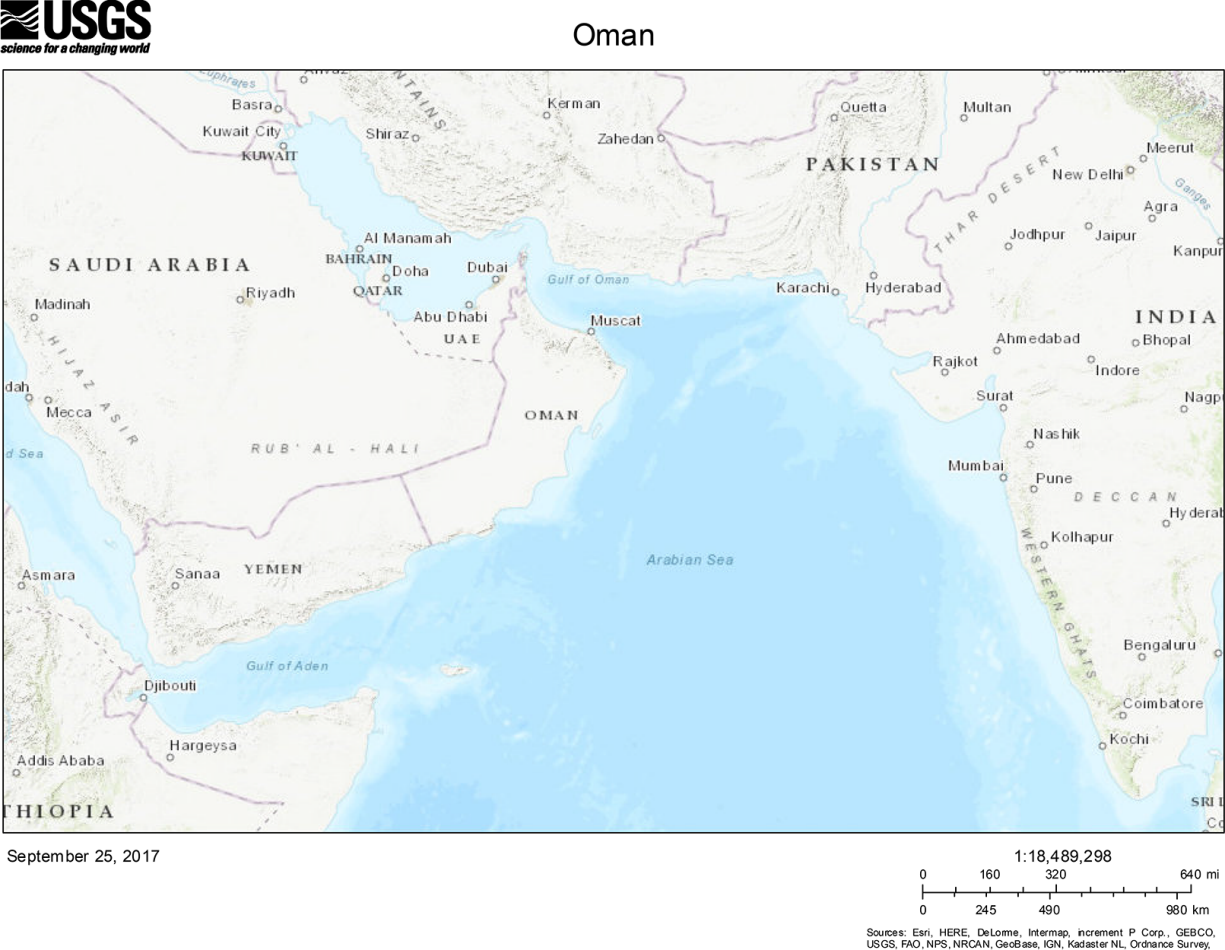
Map of Oman. doi:http://glovis.usgs.gov/.

Oman is one of the twenty-two countries in the World Health Organization (WHO) Eastern Mediterranean Region (EMR). It is located in the southeastern corner of the Arabian Peninsula with a coast that extends 3,165 kilometers from the Strait of Hormuz. Oman’s borders include Yemen to the south, and the Kingdom of Saudi Arabia and United Arab Emirates to the west (Fig 1).

The report is case record reviewing of the leprosy patients registered data since the launch of the surveillance system in 1992, to 2015. In this report, the leprosy situation will be described in detail between 2005 and 2015. Data were collected from annual reports of the Ministry of Health (MOH) [7]. Annual MOH progress reports, Ex Directorate General of Health Affairs (DGHA) and Community Health & Diseases Surveillance Newsletter, DGHA, MOH including demographics (ie age, sex, residency and nationality), new number of cases detected, relapse, contact tracing, defaulters, childhood leprosy (≤14 years), WHO grades of disability (GD) (0, 1, 2 and unknown) used and multidrug therapy (MDT) outcome including treatment completed, calculated only for nationals as almost all foreign-born population leave the country to complete their treatment at home.

Incidence of leprosy per 10,000 population, disability rate per million population of new cases, and targets including zero grade-2 disabilities (G2D) among children diagnosed with leprosy and the reduction of new leprosy cases with G2D to <1 case per million population were calculated based on WHO indicators[1,3].

The national population used to calculate leprosy indicators reported by the National Center for Statistics and Information [7]. The data analysis was conducted using Epi-Info 6 software and p value < 0.05 was considered as a cut off point for significance of the association using Chi-square and two tailed t-test.

### Disease surveillance

In March 1991, the National Communicable Disease Surveillance System was formally launched and the Department of Communicable Disease Control, functions as the apex body. The national surveillance system ensures the collection and use of appropriate and timely data for dealing with the target priority diseases. Governmental public health specialist are assigned at each governorate to oversee the different surveillance activities [6, 8].

Leprosy reporting is integrated into the National Communicable Disease Surveillance System, under group B. The surveillance monitors leprosy incidence, prevalence along with other notifiable vaccine-preventable diseases.

### Case ascertainment

**Fig 2:**
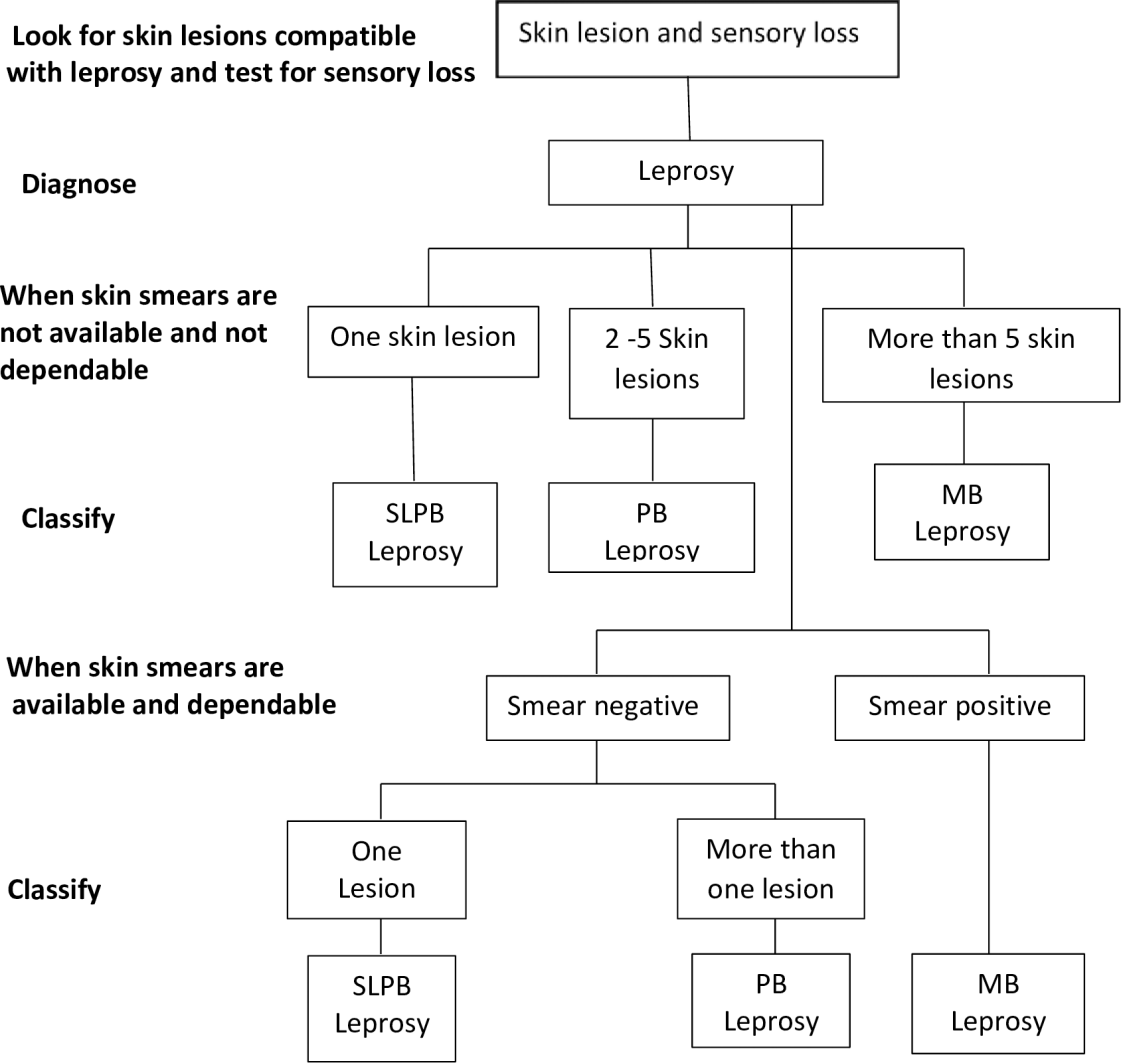
The disease leprosy classification algorithm, Oman. SLPB: Skin lesion pauci-bacillary (PB) and multi-bacillary (MB).

Both active and passive leprosy surveillance were adopted nationally, maintained and intensified in Oman. The disease leprosy is classified based on WHO case definition [9] as pauci-bacillary (PB) or multi-bacillary (MB) on the basis of clinical examination of the patient and slit skin smear. A leprosy case is diagnosed when more than five skin lesions or at least two enlarged peripheral nerves or positive of acid-fast bacilli slit skin smears is named multi-bacillary (MB) case, whereas more than five skin lesions and no more than one enlarged peripheral nerve and no presence of acid-fast bacilli in positive slit skin smears is named pauci-bacillary (PB) case (Fig 2). The skin biopsy is reviewed by leprologist.

**Fig 3:**
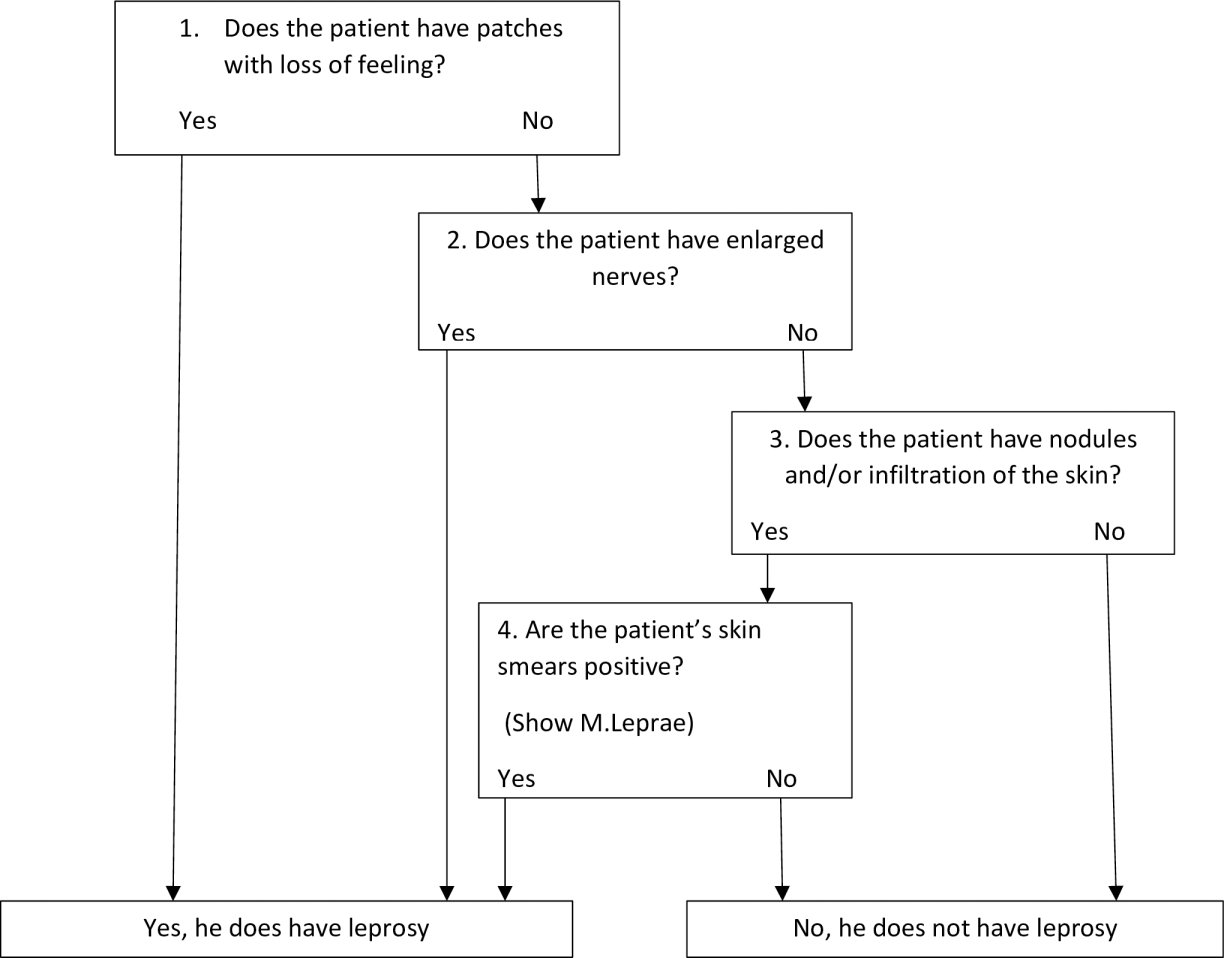
Screening of suspected cases algorithm, Oman.

The suspected patient is usually screened based on symptoms and sign as per the algorithm [Fig 3]; once confirmed as suspected referred to the nearest dermatology clinics close to the patient’s residential area. The MOH leprosy reporting policy requires all health institutions, including non-MOH (private) institutions, to notify suspect and confirmed leprosy cases as early as possible and within 7 days of suspicion to the nearest assigned governorate Communicable Diseases Surveillance Control Unit (CDSU). Leprosy reporting policy also requires to report zero monthly reporting of leprosy from all reporting institutions.

### Testing and management

For every suspected case a clinical evaluation, skin smears and/or biopsy are performed at a dermatology clinic. Samples are tested using slit skin smear for *Mycobateria leprae* at the dermatology specialized clinic based on WHO recommendations [9] and national guidelines.

The program adopted a policy of giving MDT regimens as recommended by WHO [9] for PB and for MB cases. Leprosy cases (smear positive and negative) are managed and followed-up at secondary and tertiary care dermatology clinics where medicines are offered free of charge including foreign–born residents. The diagnosis of leprosy cases is based on clinical signs and/or skin smear and biopsy and all cases are evaluated, assessed for disability and treated by leprologist. The patients are also assessed during subsequent visits for relapse, and long-term leprosy disability.

### Leprosy counseling

All patients are offered pre-test counseling covering the health implications of leprosy, and post-test counseling services after test results are ready.

### Contact tracing and follow-up

All close household contacts of an indexed leprosy case are promptly screened and followed-up annually up to 5 years.

### Leprosy monitoring system

The governorate Communicable Disease Surveillance Unit is collecting and compiling monthly summaries from dermatology clinics of both public and non-public (private) providers. These include number of total positive leprosy cases, residence, sex, age, nationality, residential and close contacts details. Monitoring also includes adherence, treatment failure, severe reactions, complications and possible toxicities of MDT, and record of leprosy-related disabilities. Such data are disaggregated from subunits to the central level on a monthly base.

The data entry is carried out at the dermatology clinic level where there is adequate human resources to ensure timely and effective leprosy data entry. The governorate surveillance unit is ultimately forwarding the aggregate data to central department of surveillance on a monthly base.

Furthermore, the leprosy-positive cases are usually followed and monitored at the dermatology clinics at secondary or tertiary care, and contact tracing and screening of all close contacts, especially household contacts, is also conducted by the governmental epidemiologist or sanitary inspector at the PHC.

Training of staff at PHC and dermatology clinics level are being conducted regularly to ensure a standard knowledge, suspect and diagnosis of leprosy case as well as reminding then about the requirement for timely reporting of the leprosy cases especially at non-MOH institutions. The MOH governorate Communicable Diseases Units regularly forwards communications out to all PHC physicians reminding them about the requirement for timely and zero reporting of leprosy cases. In addition, regular technical supervision and knowledge are updated.

The leprosy program at central level oversees the progress of the program, compiling and aggregating the national monthly reports of the leprosy cases, contact screening and defaulter retrieval reports and produces an annual report.

### Community action

Regular series of articles, interviews and media programs are delivered to the community, specifically to the patients, their families and contacts as well as among foreign-born community, for motivating people to seek for medical advice and treatment as well as minimizing the discrimination.

## Results

**Fig 4:**
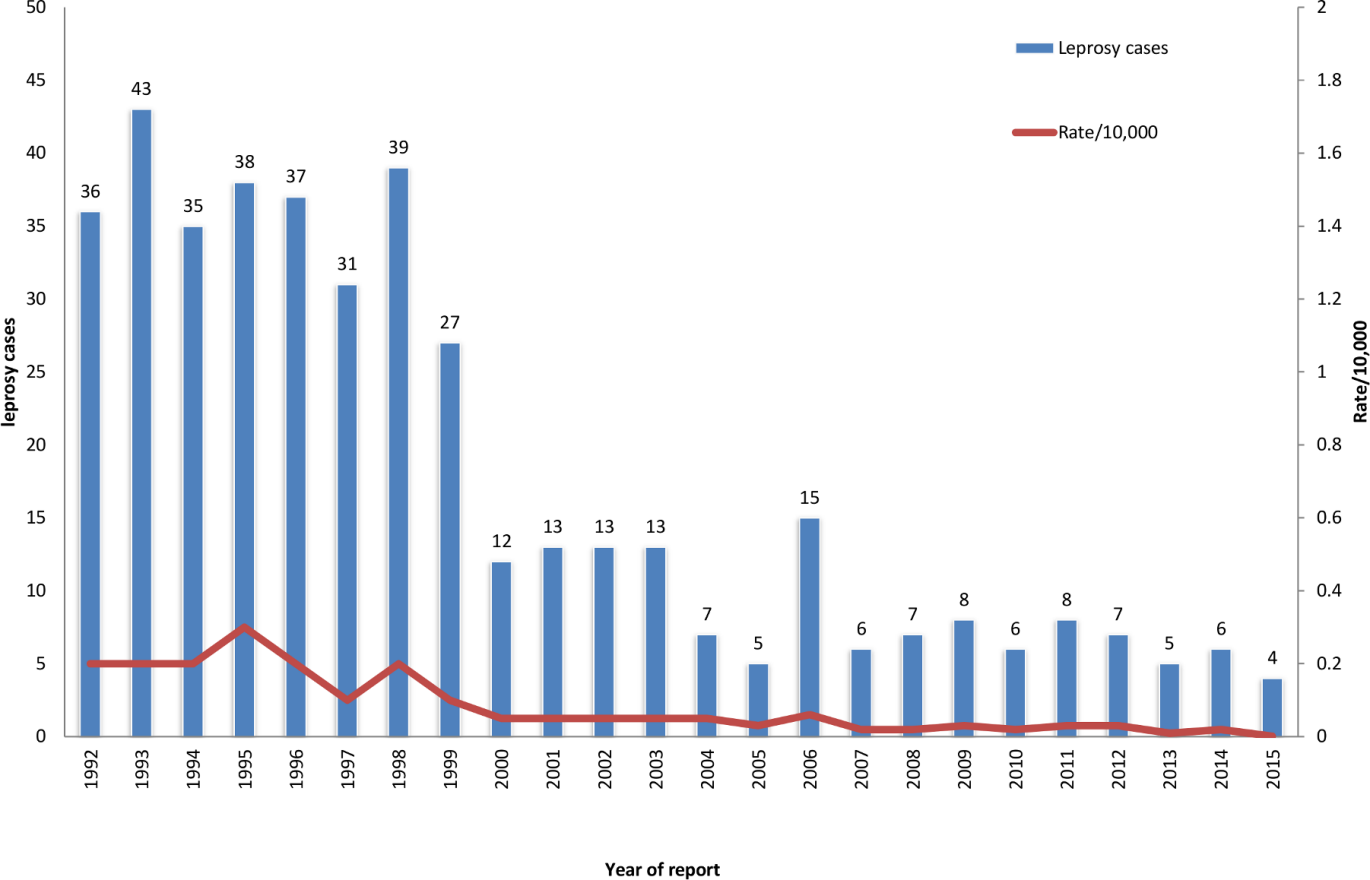
Leprosy cases and case detection rate 10,000 population by year of report, Oman, 1992–2015.

The leprosy prevalence rate significantly declined from 1.64 to 0.09 per 10,000 population during the period 1992 to 2015, (p<0.0001, Chi-square = 136.06). The prevalence of leprosy has also declined from 35 to 40 cases (rate ranged from 1.64 to 0.52 per 10,000 population) during 1992 to 1999, and further declined sharply to 13 from 4 cases (rate ranged from 0.05 to 0.001 per 10,000 population) between 2000 and 2015 (Fig 4). Based on WHO definition (rate < 1/10,000 population), Oman achieved elimination target since 1996 and maintained it thereafter (Fig 4).

The overall decline in the leprosy prevalence rate was 99%, with the heaviest reduction took place from 1992 to 2000.

From 2005 to 2015, 77 patients met the definition of leprosy in the country. Of these, 53 (69%) cases were among foreign-born (non-national) versus Oman national (p<0.003) (Table 1). 37/53 (70%) were among those who recently arrived in the country and short term workers (6–8 months).

**Table 1:** Leprosy cases disease description, Oman, 2005–2015.

Nationality	Total		Nationals n(%)		Foreign-born n(%)		p value
**Leprosy cases**	**77**		24 (31)		53 (69)		0.003 Nationals versus Foreign-born
**Age**							
0–14	6(8)		6 (19)		0		
15–19	1 (1)		1 (4)		0		
20–24	4 (5)		1 (4)		3 (6)		
25–34	24 (31)		3 (13)		21 (40)		
35–44	13 (17)		4 (16)		9 (17)		
45–54	25 (32)		7 (23)		18 (34)		
55–64	4 (5)		2 (6)		2 (4)		
>65	0		0		0		
**Children ≤ 14 years representation**	6/77 (8)		6 (19)		0		
**Sex**	**Male**	**Female**	**Male**	**Female**	**Male**	**Female**	
	58/77 (75)	19/77 (25)	15/24 (63)	9/24 (37)	43/53 (81)	10/53 (19)	0.001 for foreign-born
**Residence**							
Muscat	29/77 (38)						0.03 Muscat versus rest of Oman
North Batinah	15/77 (19)						

Out of the total, 40% were among age group 25–34 years, and 52% were above 35 years, of which 81% were male and single (Table 1). Of those, 29/77 (38%) reside in Muscat Governorate (the capital) followed by North Batinah Governorate 15/77 (19%). Out of the total leprosy cases, 75/77 (97%) had positive smear or biopsy [Table 1].

**Fig 5:**
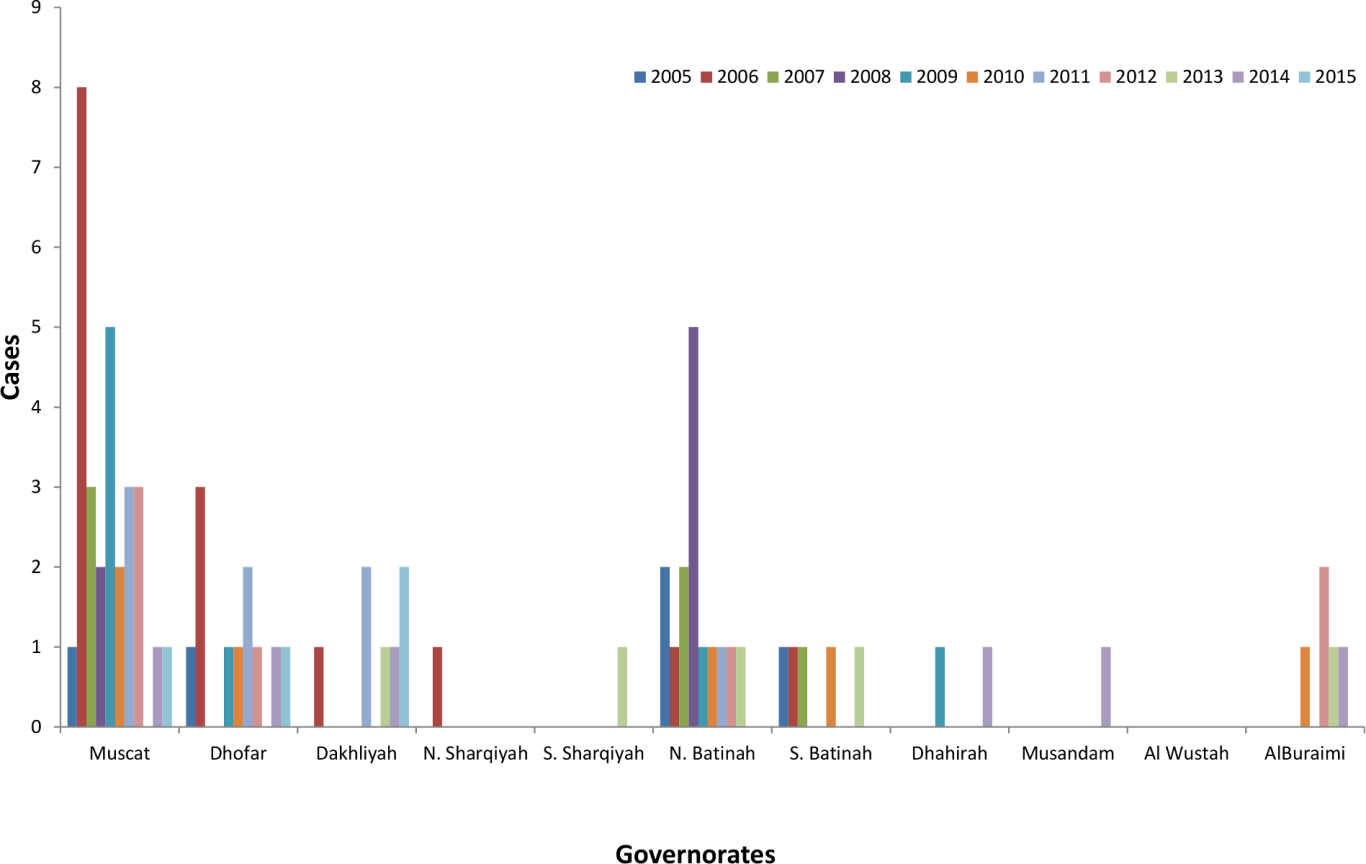
Distribution of leprosy cases by governorate (provinces), Oman, 2005–2015.

Women with leprosy accounted for 19/77 (25%) of the total cases. There has been zero leprosy case among national woman since 2013. Most of the leprosy cases were notified in Muscat governorate 29/77 (38%) (Average rate 0.3 per 10,000) (p*<* 0.03, Muscat versus the rest of the Governorates) and north Batinah Governorate 15/77 (19%) (Average rate 0.3 per 10,000) as shown in Fig 5.

Patient’s ≥ 45 years of age represented the largest group of cases 29/77 (38%), followed by age 25–44 years group 24/77 (31%) (Table 1).

**Fig 6:**
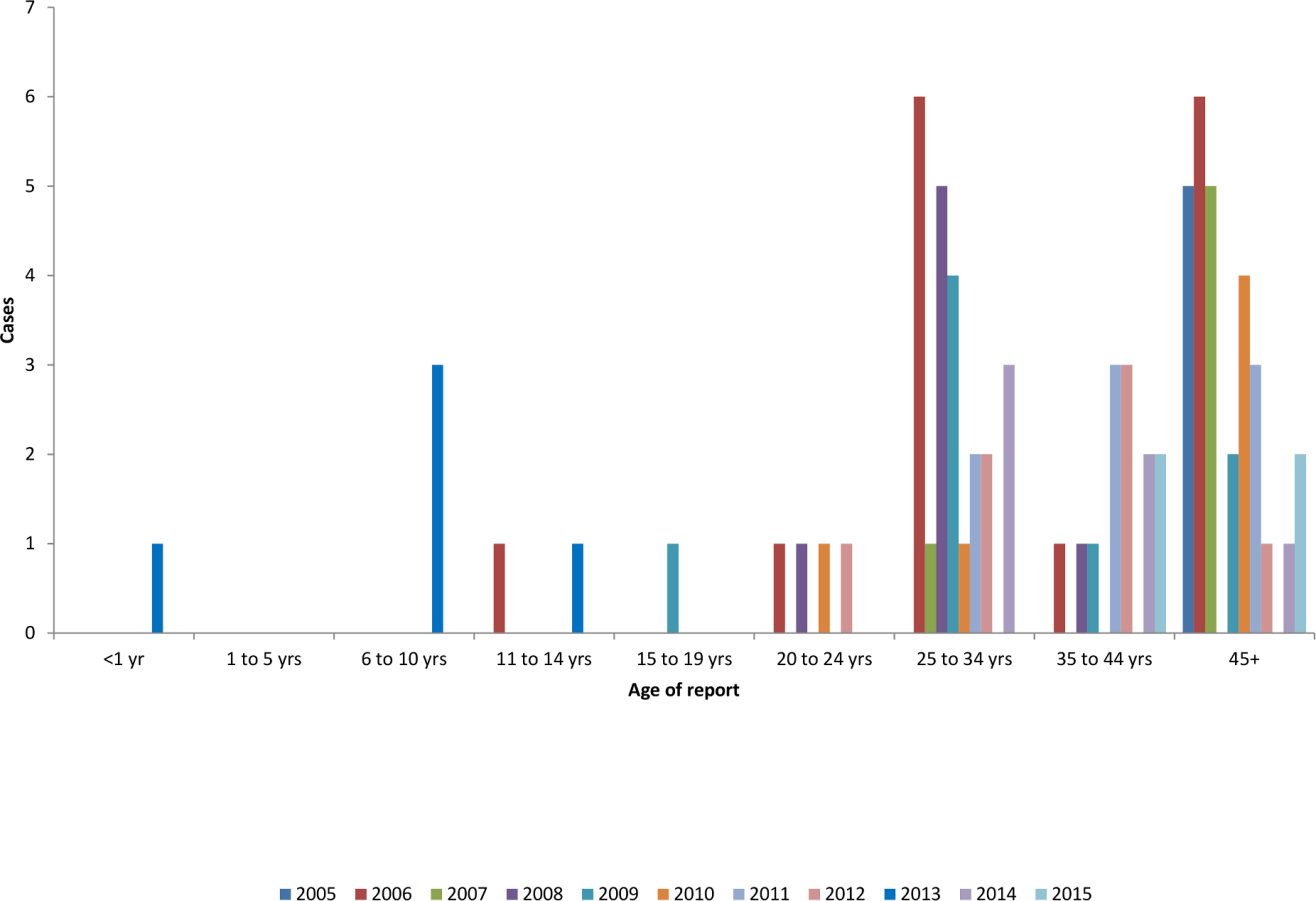
Leprosy cases by age group, Oman, 2005–2015.

During the same period (2005 to 2015), children less than 14 years represented 6/77 (8%) of the total leprosy cases (Table 1), all were identified during active search of the contact tracing and were reported before the year 2014 (Fig 6). The age distribution of the cases were 1/24 (4%), 3/24 (13%) and 2/24 (8%) among < 1 year, 6–10 and 11–14 years respectively. No cases < 14 years among foreign-born. No disabilities were reported among newly diagnosed children since 2005.

Out of the total, 60 (78%) and 17 (22) leprosy cases were MB and PB respectively (p<0.01). Further, from 2005 to 2015, 24 (31%) leprosy cases were among national, 15/24 (63%) and 9/24 (37%) were male and female respectively.

Out of the total national leprosy cases, 20/24 (83%) and 4/24 (17%) were MB and PB cases, respectively (Table 2). The average number of MB cases per year is 2, and the range varied from 2 to 3 cases. The MDT completion rate reached almost 100% and no relapse or defaulter cases were notified. Of the total cases, 3/77 (44%) were reported with hands and/or feet disability, all were among nationals. 0/3 (0%) had grade-0 disability (G0D), 0/3 (0%) grade-1 disability (G1D), and 3/3 (100%) grade-2 disability (G2D) upon diagnosis. The rate of new patients diagnosed with leprosy related disability was approximately 0.9 per million population, and G2D rate among nationals was 0.9 per million population. No disability were recorded among woman or children (≤14 years) national residents and foreign-born residence by year 2013 and onward (Table 2).

**Table 2:** Leprosy disease classification, multidrug therapy (MDT) and disability, Oman, 2005–2015.

Nationality	National’s n (%)	Foreign-born (non-nationals) n (%)	P value
**Disease classification**			
Pauci-bacillary (PB)	4 (17)	13 (25)	0.01for total MB (60)versus PB (17)
Multi-bacillary (MB)	20 (83)	40 (75)	
Multi drug therapy (MDT)completion rate	24 (100)	Unavailable	
**Relapse or defaulter**	Nil	Unavailable	
**Total leprosy cases reported with hands and/or feet disability at diagnosis**	3/77 (4)		
	3/24 (13)	0/53	
**Grade of disability**			
Grade-0 disability (G0D)	**0**	0	
Grade-1 disability (G1D)	0	0	
Grade-2 disability (G2D)	3/3 (100)	0	
**Leprosy related disability**	0.9 per million population	0	
**Grade 2 disability (G2D) rate**	0.9 per million population	0	

While 53 (69%) leprosy cases were among foreign-born (non-national), 43/53 (81%) were male (p<0.003). Out of the total national leprosy cases, 13/53 (25%) and 40/53 (75%) were MB and PB cases, respectively (Table 2). The MDR completion and cure rate couldn’t be calculated for foreign-born as almost all cases leave for their country of origin to complete the treatment.

Neither data reported on screening of close contacts especially household contacts among leprosy cases published nor surveillance for antibiotic resistance to MDT established.

### Management of complications and prevention of disabilities

The MOH set-up good records to keep track of leprosy patients in order to provide appropriate post treatment care to prevent and manage residual post-treatment disabilities. These supports are provided free of charge by the MOH institutions and other related ministries.

## Discussion

By 2015, Oman has achieved elimination as a public health problem goals (Target< 1/10,000 per population) by implementing the WHO global elimination main strategies [2] including achieving and sustaining good national surveillance system, integration into other disease communicable control program which facilitated supervision and monitoring of leprosy program as well as maintaining inclusive high universal coverage. As a result of implementing these strategies, leprosy incidence rate decreased by 99% since 1992. Oman has thus successfully eliminated Leprosy by WHO standards. The next goal is to achieve a zero-leprosy state. The drop of leprosy cases from 1998 to 2000 may be attributed to immigration policy, where the majority of the foreign-born were from India.

Oman also made impressive progress towards becoming a leprosy-free country to meet the key targets [2]; Firstly, no leprosy cases among age group ≤14 years since 2013 and zero disabilities reported among newly diagnosed children (Target zero G2D) [9]. Similar findings were reported from 39-member states zero new G2D among children cases [9]. Secondly, the incidence of leprosy declined to < 1 case per million population by 2015 and thirdly, the implementation of free and universal coverage of MDT as well as providing management of complications and prevention of disabilities with focus on children, women and all population indiscriminately.

Similar findings reported by WHO, out of the total member states, 62 informed on foreign-born patients, out of those, 44-member states reported zero cases while 18 reported 743 foreign-born cases being treated in their respective national leprosy program [4]. Oman is among few countries reporting leprosy among foreign-born patients as well as providing leprosy diagnosis and treatment free of charge without discrimination. Furthermore, Oman has a high population of foreign-born (non-Omani) residence workers and their families (44%), out of which 85% are male and 92% are above age of 20 years and 70% of the foreign-born are coming from India, Bangladesh and Pakistan [7]. Low proportion of female in new cases among foreign-born (19%) reported is reflective of high population of male work force (85%) [7]. The foreign-born population frequently travels in and out from high leprosy endemic countries. The possible reasons for foreign-born patients leaving the country shortly after diagnosis of leprosy were the very short term contract (6 to 8 months), discretionary employment practices by the employers and the preference of patients to go home to complete their leprosy treatment. In order to facilitate reducing the incidence as well as to minimize delay in identification of leprosy cases among foreign-born population, currently the Executive Board of the Health Ministers' Council for Cooperation Council States [10] adopted screening policy on leprosy during pre and post arrival for foreign-born population. In the future, the program should also undertake and reinforce screening procedures upon arrival to the country, and in private sectors institutions where most of the foreign-born populations seek medical advice in order to detect leprosy early and to provide MDT at the earliest instance, which remains the fundamental principles of leprosy control. Oman has achieved leprosy elimination since 1996, but is still reporting low number of leprosy cases among national 24 (31%), the program therefore needs to review each case in-depth to find out the possible source of infection especially the possibility of close contact with foreign-born house help who might having undiagnosed leprosy among other possibilities of contamination.

The study findings showed no detection of leprosy cases among children ≤14 years since 2014, reflecting negligible or low transmission of the disease in the community which are reflected by overall low leprosy prevalence (< 1/10,000 per population), and cases notified between 2005 to 2015 especially among nationals (31%). The study showed MDR completion and cure rate reached almost 100% and no relapse among national cases indicating treatment adherence and completion by the patients and good access to health services across the country. The high zero proportion of patients with G0D and G1D among newly diagnosed leprosy patients indicates and reflects the efficiency of early detection of leprosy and high awareness of leprosy by the health staff. While the high proportion of patients with G2D among newly diagnosed leprosy patients (100%) reflects the delay in detection, diagnosis or leprosy patients receiving inappropriate heath care services. However, this finding was higher than the average G2D rate of leprosy in China (25.4%) [11].

The risk of acquiring leprosy for close contact living in same households with MB and PB patients is almost 5–10 and 2–3 times higher respectively, compared to people not living in such households. Therefore, unrecognized leprosy cases and subclinical infections among household contacts contribute a significant proportion of overall new leprosy cases [12]. Our study showed high proportion of MB cases for both nationals (83%) and foreign-born (75%) reflecting delay in detection of leprosy in the community. Therefore, the national leprosy control program is to focus on promoting early detection monitoring thorough measurement of disability in new cases and conducting prompt investigation looking at reasons for delay in detection, identifying the source of infection, and reviewing the mandatory contact tracing programs. These steps would undoubtedly reduce the disease burden. Investigating for stigma as an important cause of delayed diagnosis facilitating transmission of infection with communities is also important. Additionally, national efforts are also needed to involve dermatologists in private sector to sustain high-quality leprosy services especially providing treatment.

Although many member states declared leprosy elimination at the national level, leprosy as a public health problem has remained at sub-national levels [13–15], and many other leprosy concerns were unrecognized and unresolved [16, 17]. In our study we found that Muscat and north Batinah Governorates are reporting highest leprosy cases, these two urban governorates are the most populated governorates and comprise of 49% of the total population with highest population density 346 and 87 /square kilometer in Muscat and North Batinah Governorates respectively, therefore, implementation of targeted active case search in high-risk governorates is required.

The program is to introduce surveillance for MDT antimicrobial resistance as well.

The progress made towards a leprosy-free Oman has been facilitated by several factors including strong political commitment demonstrated by a leprosy program that is more than 30 years old, good universal coverage of MDT which may be due to free management of leprosy cases with all residents and well-trained governorate dermatologist across the country. Additional factors include counselling services provided to the leprosy patients.

Oman is committed to the global leprosy-free goal and have achieved leprosy-free targets among those mainly zero G2D among paediatric leprosy and reduction of G2D to close to 1/1,000,000 population by 2015. However, Oman also needs to embark on rigorous methods to evaluate the program at grass root to identify the potential areas for improvement towards zero incidences of leprosy, and update the national guidelines to meet the new global WHO strategies as well as establish leprosy validation and verification task force aimed at oversight and coordination efforts among different stakeholders. The current study has some limitations including missing MDT completion rate, calculation of G2D to <1 case per million population wasn’t calculated and information collected on country of origin, duration between entering the country and initial diagnosis and proportion of household contacts screened among leprosy cases weren’t available among foreign-born and nationals population.

In conclusion, Oman has met the elimination as a public health problem goal and made great strides towards becoming a leprosy-free country however, challenges remain with regards to sustaining these achievements and moving towards becoming a leprosy free-country. The National Leprosy Control Program should focus efforts in the near future to reduce the burden of leprosy to zero by maintaining and improving surveillance system efficiently and increase sensitivity for timely detection of leprosy cases. The program is to also establish an independent verification committee to reinforce screening procedures among foreign-born population regularly review the status of leprosy-free patients and provide advice on corrective actions. These steps will prove critical to achieving and maintaining successful zero leprosy case.

## Supporting information

S1 Checklist STROBE checklist. (DOC)
